# TAB182 regulates glycolytic metabolism by controlling LDHA transcription to impact tumor radiosensitivity

**DOI:** 10.1038/s41419-024-06588-8

**Published:** 2024-03-13

**Authors:** Shi Chen, Da-Fei Xie, Saiyu Li, Jinhua Luo, Yang Han, Hejiang Guo, Shuaining Gao, Xin Huang, Hua Guan, Ruixue Huang, Ping-Kun Zhou

**Affiliations:** 1 School of Public Health, Hengyang Medical School, University of South China, Hengyang, Hunan Province 421001 P. R. China; 2 Department of Radiation Biology, Beijing Key Laboratory for Radiobiology, Beijing Institute of Radiation Medicine, Beijing, 100850 P. R. China; 3 School of Life Sciences, Hebei University, Baoding, Hebei Province 071002 P. R. China; 4 Department of Occupational and Environmental Health, Xiangya School of Public Health, Central South University, Changsha, Hunan 410078 P. R. China

**Keywords:** Transcriptional regulatory elements, Radiotherapy

## Abstract

Metabolic reprogramming, a hallmark of cancer, is closely associated with tumor development and progression. Changes in glycolysis play a crucial role in conferring radiation resistance to tumor cells. How radiation changes the glycolysis status of cancer cells is still unclear. Here we revealed the role of TAB182 in regulating glycolysis and lactate production in cellular response to ionizing radiation. Irradiation can significantly stimulate the production of TAB182 protein, and inhibiting TAB182 increases cellular radiosensitivity. Proteomic analysis indicated that TAB182 influences several vital biological processes, including multiple metabolic pathways. Knockdown of TAB182 results in decreased lactate production and increased pyruvate and ATP levels in cancer cells. Moreover, knocking down TAB182 reverses radiation-induced metabolic changes, such as radioresistant-related lactate production. TAB182 is necessary for activating LDHA transcription by affecting transcription factors SP1 and c-MYC; its knockdown attenuates the upregulation of LDHA by radiation, subsequently suppressing lactate production. Targeted suppression of TAB182 significantly enhances the sensitivity of murine xenograft tumors to radiotherapy. These findings advance our understanding of glycolytic metabolism regulation in response to ionizing radiation, which may offer significant implications for developing new strategies to overcome tumor radioresistance.

## Introduction

Radiation resistance is a significant obstacle to cancer radiotherapy. It is reportedly influenced by tumor-specific oncogenic signaling pathways, cancer cell proliferation potential, genetic or intrinsic mechanisms of cancer cells, and the tumor microenvironment, including hypoxia [1]. Despite extensive studies on tumor radiation resistance, it remains a formidable barrier to effective tumor treatment. Therefore, elucidating the mechanisms underlying tumor radioresistance and enhancing tumor radiosensitivity are imperative for advancing malignancy treatment. Molecular mechanisms contributing to radiation resistance of tumor cells include (1) Alterations in DNA repair mechanisms [2], where tumor cells may exhibit disrupted or modified DNA repair mechanisms, increasing their resistance to radiation damage; (2) Changes in cell cycle distribution [3], where tumor cells may alter their cell cycle progression, becoming more likely to enter the radiation-resistant G0 phase; (3) Alterations in apoptosis mechanisms [4], where tumor cells may have defects or modifications in apoptosis mechanisms, increasing their resistance to radiation-induced apoptosis; (4) Changes in oxidative stress response [5], where tumor cells may modify their oxidative stress response, with increased antioxidant capability leading to increased resistance to radiation damage; (5) Metabolic reprogramming, where resistance to radiotherapy can be acquired through enhanced glycolysis and metabolic adaptations. Consequently, radioresistance represents a multifactorial phenomenon occurring in diverse cells through various regulatory mechanisms involving different molecules.

In cancer cells, metabolism markedly differs from normal cells, most notably in that tumor cells derive more energy from enhanced glycolysis and truncated tricarboxylic acid cycle to produce ATP. They utilize amino acids, fatty acids lactate, and so on as precursors for ATP production and biosynthesis, meeting their nutritional and energetic needs for rapid growth and division [6, 7]. Additionally, ionizing radiation has been found to induce metabolic disorders in cells, including energy metabolism disorders [8], characterized by reduced ATP synthesis, affecting cell growth and differentiation; protein metabolism disorders [9, 10], impacting protein synthesis and degradation, influencing cell growth and differentiation; lipid metabolism disorders, altering lipid synthesis and degradation, affecting cell growth and differentiation; carbohydrate metabolism disorders [11, 12], influencing carbohydrate metabolism, impacting cell growth and differentiation; and amino acid metabolism disorders [12], affecting amino acid metabolism, impacting cell growth and differentiation. These metabolic disorders create an imbalance in the synthesis and degradation of biological molecules, affecting normal metabolism and cell growth. Furthermore, some studies have shown that radiation exposure may facilitate metabolic reprogramming in tumor cells, enhancing their survival ability and resistance to treatment [12, 13]. Thus, understanding the role of ionizing radiation in the metabolism of cancer cells is crucial for comprehending their metabolic reprogramming mechanism during radiotherapy, including metabolic pathway alterations and metabolic product accumulation, providing insights into radiotherapy resistance. Targeted interventions like metabolic therapy and nutritional intervention, informed by this understanding, can enhance tumor cell radiosensitivity and improve radiotherapy efficacy.

Tankyrase 1 binding protein 1 (TAB182) is a regulatory factor involved in cell invasion, DNA repair, and telomere stability, with functions including cell cycle regulation, DNA repair promotion, and telomere length regulation [14–18]. Research has demonstrated a strong correlation between TAB182 and radiation sensitivity. TAB182 expression is higher in tumor tissues compared to non-carcinoma counterparts, correlating with poor prognosis in radiotherapy [14]. Additionally, radiation exposure has been shown to increase TAB182 expression. TAB182 is involved in DNA damage repair, and its knockdown leads to reduced DNA damage repair efficiency [19]. However, the role of TAB182 in metabolism remains underexplored. Previous work revealed significant changes in glucose metabolism-related protein levels in cell lines with TAB182 depletion. Experimental validation showed a marked decrease in lactate production levels in cell lines with TAB182 knockdown. Combining this with radiation treatments, it was found that TAB182 knockdown enhances tumor cells’ radiation sensitivity. This suggests that exploring TAB182’s role in metabolism offers a new perspective for overcoming radiotherapy resistance.

## Results

### TAB182 is related to the determination of radiation sensitivity

**Fig. 1:**
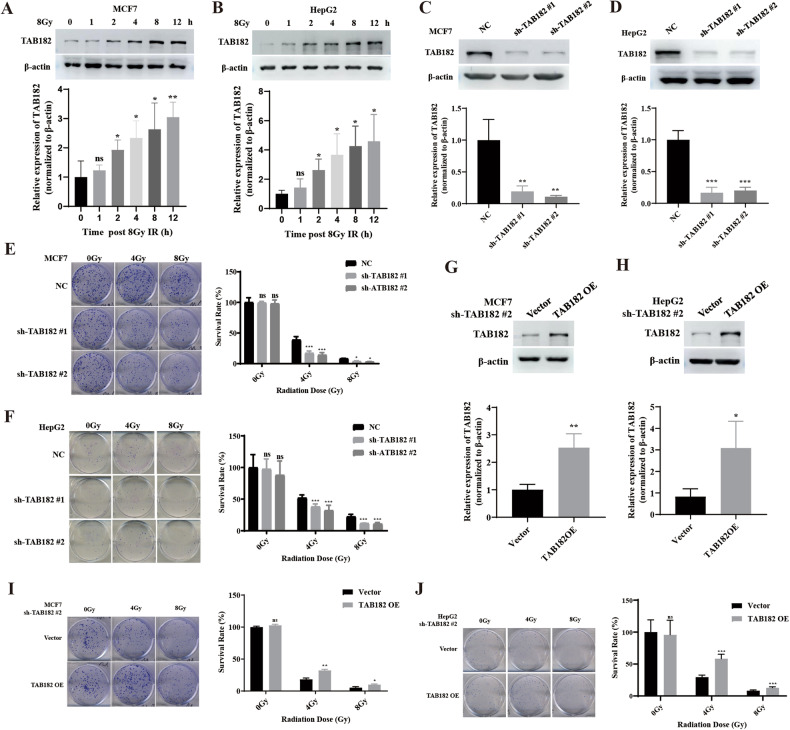
TAB182 and radiation sensitivity. **A**, **B**. TAB182 levels gradually increased in MCF7 and HepG2 cells after 8 Gy radiation. **C**, **D** Western blot validation confirmed TAB182 knockdown in MCF7 and HepG2 cells. **E**, **F** Clonogenic assay revealed increased radiation sensitivity in tumor cells after TAB182 knockdown. **G**–**J** Reintroducing TAB182 in the TAB182 knockdown cell lines enhanced the radiation resistance of tumor cells. Data represent means ± SDs from three independent experiments. *P < 0.05; **P < 0.01; ***P < 0.001.

In MCF7 and HepG2 cells, a progressive increase in TAB182 expression was observed after exposure to 8 Gy of cobalt-60 γ-ray irradiation, indicating a significant link between TAB182 and radiation response in tumor cells (Fig. 1A, B). Therefore, lentivirus-mediated stable TAB182 knockdown was employed in MCF7 and HepG2 cells to analyze TAB182’s function in tumor cell radiosensitivity (Fig. 1C, D). The results showed that in the TAB182 knockdown group, tumor cells exhibited a significantly lower colony formation rate after exposure to 4 Gy and 8 Gy radiation compared to the control group, indicating that TAB182 knockdown enhances radiation sensitivity in tumor cells (Fig. 1E, F). Additionally, a TAB182 rescue experiment in the TAB182 knockdown cell lines (Fig. 1G, H) revealed that reintroducing TAB182 could reverse the increased radiation sensitivity caused by TAB182 knockdown (Fig. 1I, J). This highlights the relationship between TAB182 and radiosensitivity in tumor cells and suggests that TAB182 upregulation induced by irradiation may contribute to radiation resistance in tumor cells.

### TAB182 impacts multiple cellular processes and is crucial in glycolytic metabolism

**Fig. 2:**
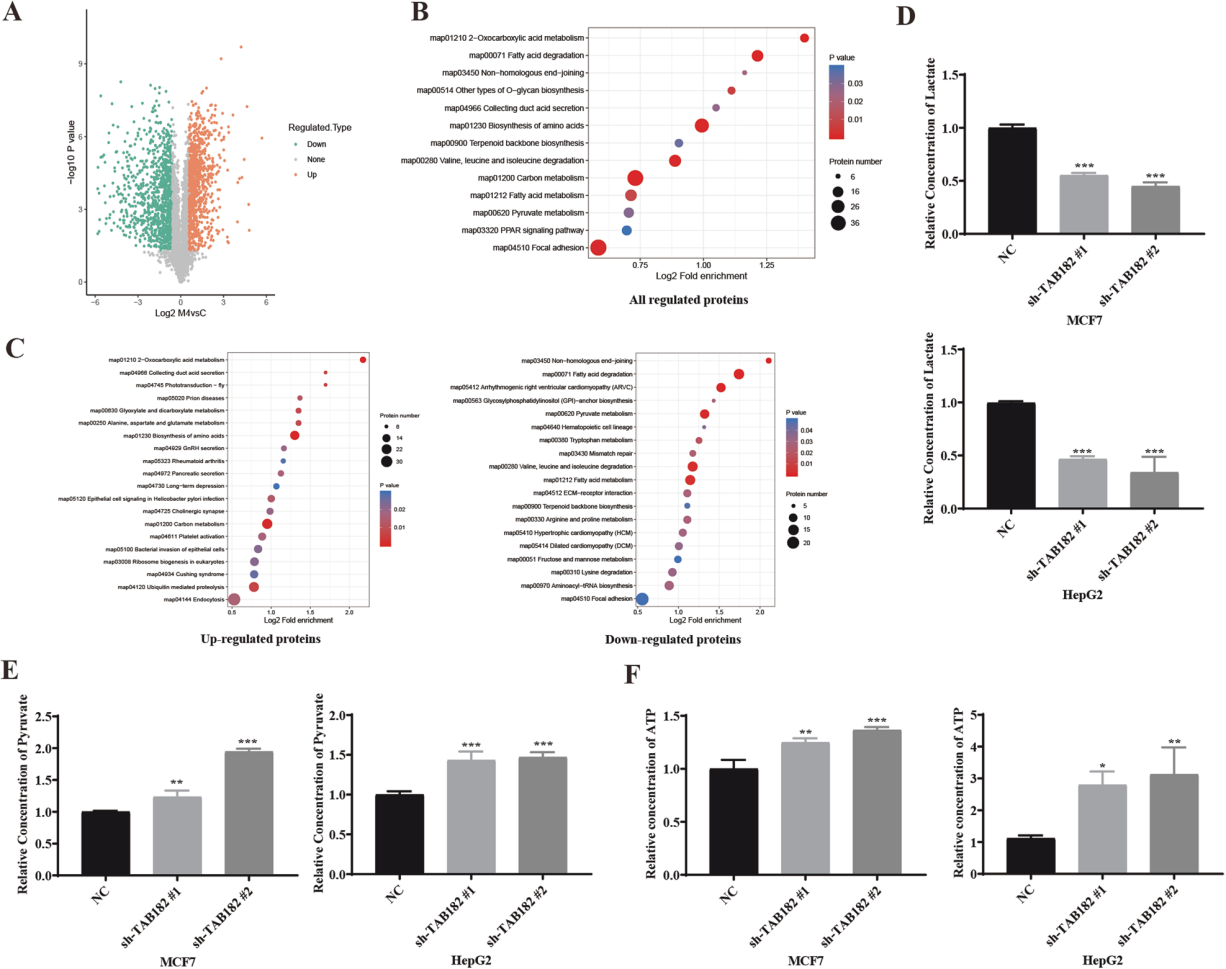
TAB182 impacts multiple cellular processes and plays a crucial role in glycolytic metabolism. **A** Volcano plot shows differentially expressed genes before and after knocking down TAB182 in MCF7 cells. **B**, **C** KEGG pathway enrichment analysis explores functional roles of regulated proteins and subsets in various pathways. **D**–**F** TAB182 knockdown reduces lactate and increases pyruvate and ATP in MCF7 and HepG2 cells. Data represent the means ± SDs from three independent experiments. *P < 0.05; **P < 0.01; ***P < 0.001.

To analyze the function of TAB182 in biological processes of tumor cells, a proteomic analysis was conducted on TAB182 knockdown MCF7 cells. With TAB182 knockdown, 890 proteins were upregulated, and 1024 were downregulated (Fig. 2A). Enrichment analysis of the regulated molecules using KEGG pathways indicated a significant relation of these proteins to carbon metabolism, biosynthesis of amino acids, and carbon metabolism (Fig. 2B). Notably, the upregulated proteins were predominantly associated with carbon metabolism and biosynthesis of amino acids pathways (Fig. 2C), and the downregulated proteins were mainly enriched in pyruvate metabolism and fatty acid metabolism pathways (Fig. 2C). These findings suggest that TAB182 influences multiple life processes in tumor cells and plays a significant role in metabolism.

Proteomic analysis revealed that TAB182 knockdown significantly impacts pyruvate and one-carbon metabolism pathways in tumor cells. This observation led to the hypothesis that TAB182 is crucial for the glucose metabolism of these cells. Consequently, intracellular lactate level measurements were conducted in MCF7 and HepG2 cells following TAB182 knockdown. The results showed a notable reduction in intracellular lactate content compared to the control group (Fig. 2D). To elucidate the effect of TAB182 on lactate metabolism, TAB182 replenishment was performed in the TAB182 knockdown cell lines (Supplementary Information, Fig. A), suggesting that TAB182 may be involved in the glucose metabolism pathway of tumor cells. Additionally, pyruvate levels, a key product of glucose metabolism, were measured, revealing a marked increase in pyruvate levels in tumor cells following TAB182 silencing (Fig. 2E). Replenishing TAB182 reversed this effect (Supplementary Information, Fig. B), further indicating TAB182’s critical role in the glucose metabolism process in tumor cells. Likely, TAB182 influences the conversion of pyruvate into lactate, leading to reduced lactate production and increased pyruvate levels. Moreover, intracellular ATP levels were measured, showing an increase after TAB182 knockdown in the cells (Fig. 2F). Replenishing TAB182 reversed this increase in ATP levels (Supplementary Information, Fig. C). These results indicate that TAB182 is essential for glucose metabolism, and its knockdown leads to a shift towards the tricarboxylic acid (TCA) cycle in tumor cells, signifying that TAB182 knockdown induces changes in tumor cell glucose metabolism.

### TAB182 knockdown reverses radiation-induced metabolic changes through affecting LDHA

**Fig. 3:**
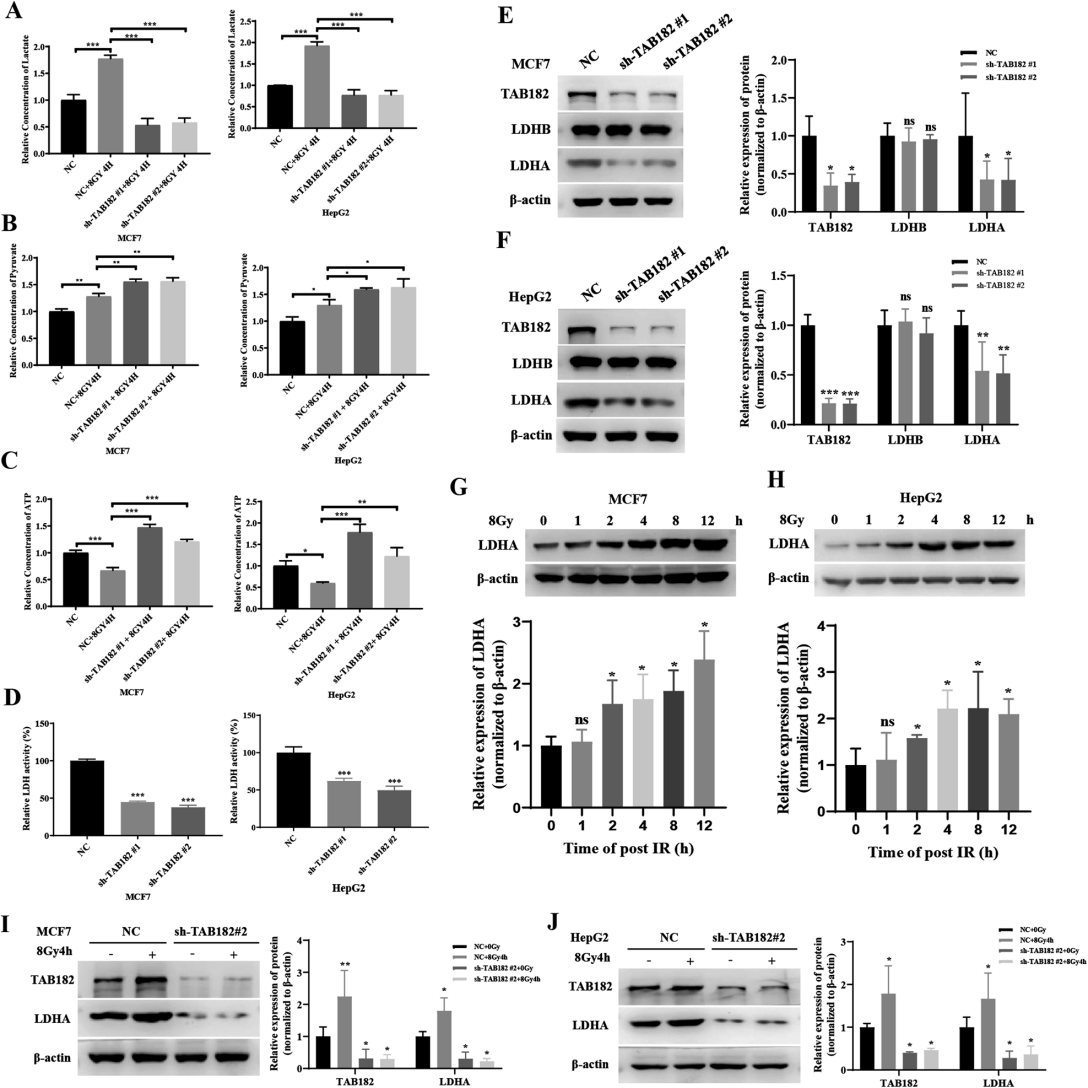
TAB182 knockdown reverses radiation-induced metabolic changes through affecting LDHA. **A** TAB182 knockdown reverses radiation-induced lactate increase. **B** Radiation and TAB182 knockdown both increase cellular pyruvate levels. **C** TAB182 knockdown reverses radiation-induced ATP reduction. **D** TAB182 knockdown reduces lactate dehydrogenase activity in MCF7 and HepG2 cells. **E**, **F** TAB182 knockdown decreases LDHA protein expression. **G**, **H** Radiation increases LDHA protein expression. **I**, **J** TAB182 knockdown reverses radiation-induced LDHA expression increase. Data represent means ± SDs from three independent experiments. *P < 0.05; **P < 0.01; ***P < 0.001.

Research has established that radiation induces alterations in tumor cell energy metabolism, resulting in increased lactate production and decreased ATP generation. In this study, it was observed that in MCF7 and HepG2 tumor cells, 4 h after exposure to 8 Gy of radiation, there was an increase in intracellular lactate and pyruvate production, along with a reduction in ATP generation. However, TAB182 downregulation was capable of reversing these radiation-induced changes in intracellular lactate and ATP levels (Fig. 3A–C). This finding highlights TAB182’s crucial role in mediating radiation-related alterations in glucose metabolism. Lactate dehydrogenase activity was measured to explore the mechanism by which TAB182 affects tumor cell glucose metabolism. The results indicated that TAB182 knockdown reduced cellular lactate dehydrogenase activity (Fig. 3D), while TAB182 replenishment reversed this decrease in activity (Supplementary Information, Fig. D). This reduction in lactate dehydrogenase activity decreased the conversion of pyruvate to lactate, explaining the decreased lactate production in tumor cells following TAB182 knockdown. Subsequently, Western blot experiments were conducted to analyze proteins associated with lactate dehydrogenase (LDH). The results showed that TAB182 knockdown notably decreased lactate dehydrogenase A (LDHA) expression, with no significant impact on LDHB expression (Fig. 3E, F). Further, TAB182 rescue experiments in TAB182 knockdown cells revealed that reintroducing TAB182 could reverse the decrease in LDHA expression caused by TAB182 knockdown (Supplementary Information, Fig. E,F). Therefore, TAB182 affects lactate metabolism by regulating LDHA expression.

Additionally, the effects of radiation on LDHA expression were explored. Following exposure to 8 Gy of irradiation, LDHA expression significantly increased in MCF7 and HepG2 cells (Fig. 3G, H). This finding suggests a correlation between radiation-induced lactate elevation and LDHA upregulation. Furthermore, the study revealed that knocking down TAB182 could effectively reverse the radiation-induced overexpression of LDHA (Fig. 3I, J). These results, which are consistent with previous lactate findings, further emphasize TAB182’s role in regulating lactate metabolism through its impact on LDHA.

### TAB182 modulates LDHA transcriptional activity through SP1 and c-MYC

**Fig. 4:**
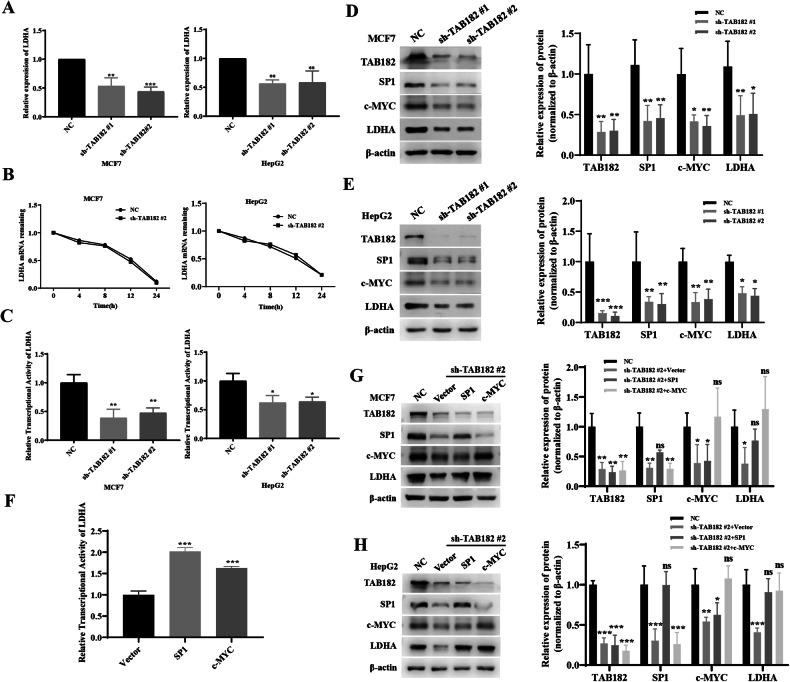
TAB182 modulates LDHA transcriptional activity through SP1 and c-MYC. **A** TAB182 knockdown reduces LDHA mRNA levels. **B** Actinomycin D inhibits de novo RNA synthesis, and LDHA mRNA stability was assessed using qPCR experiments. **C** Dual-luciferase reporter assay investigates the impact of TAB182 on LDHA transcriptional activity in MCF7 and HepG2 cells. **D**, **E** TAB182 knockdown decreased SP1 and c-MYC protein expression in both MCF7 and HepG2 cells. **F** Dual-luciferase reporter assays confirmed that both SP1 and c-MYC increased LDHA transcriptional activity in 293 T cells. **G**, **H** Western blot analysis assesses the expression levels of LDHA following SP1 and c-MYC reintroduction. Data represent means ± SDs from three independent experiments. *P < 0.05; **P < 0.01; ***P < 0.001.

To further investigate how TAB182 influences lactate dehydrogenase, qPCR experiments were conducted to measure mRNA levels of LDHA following TAB182 knockdown. The results indicated a significant reduction in LDHA mRNA expression levels due to TAB182 knockdown, suggesting that TAB182 modulates LDHA protein expression by regulating LDHA mRNA (Fig. 4A, Supplementary Information, Fig. G). To elucidate the pathways through which TAB182 affects LDHA mRNA expression, mRNA stability experiments were performed as a preliminary step. Actinomycin D was employed to inhibit de novo RNA synthesis, and LDHA mRNA levels were assessed at various points in time using qPCR. These experiments revealed that TAB182 did not significantly impact LDHA mRNA stability (Fig. 4B). Consequently, the effect of TAB182 on LDHA transcriptional activity was examined through dual-luciferase reporter experiments. These experiments showed that knocking down TAB182 significantly reduced LDHA promoter transcriptional activity, indicating that TAB182 modulates LDHA mRNA expression by influencing its transcriptional activity (Fig. 4C). To investigate how TAB182 affects LDHA transcriptional activity, a Western blotting assay was performed to examine LDHA transcription factor levels post-TAB182 knockdown. The results showed decreased SP1 and c-MYC expression following TAB182 knockdown (Fig. 4D, E). Additionally, overexpression of TAB182 can reverse the decrease in SP1 and c-MYC expression induced by TAB182 knockdown (Supplementary Information, Fig. E, F). The findings suggest that TAB182 impacts the expression of SP1 and c-MYC, potentially mediating LDHA expression through SP1 and c-MYC. To validate whether SP1 and c-MYC regulate LDHA expression, overexpression plasmids for SP1 and c-MYC were constructed. Dual-luciferase reporter assays in 293 T cells were conducted to determine if SP1 and c-MYC could modulate LDHA expression. These assays confirmed that SP1 and c-MYC increased LDHA transcriptional activity (Fig. 4F). Furthermore, replenishing SP1 and c-MYC in TAB182 knockdown cells can reverse the decrease in LDHA expression caused by TAB182 knockdown. (Fig. 4G, H). These findings imply that TAB182 may influence LDHA transcriptional activity by modulating the expression of LDHA transcription factors, thereby impacting tumor cell glucose metabolism.

### Restoring LDHA expression reverses the metabolic alterations and radiosensitivity by TAB182 knockdown

**Fig. 5:**
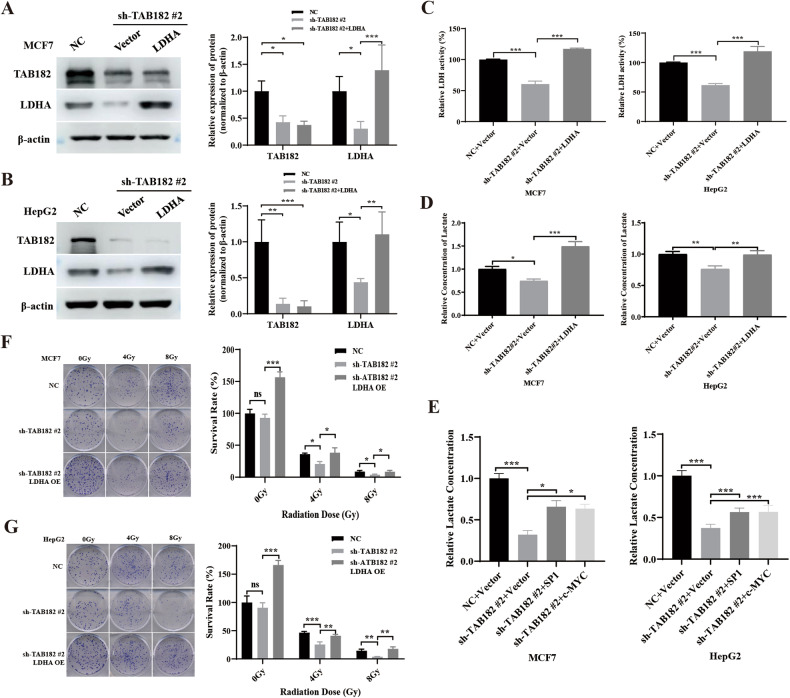
Restoring LDHA reverses metabolic alterations and radiosensitivity induced by TAB182 knockdown. **A**, **B** Western blot analysis assesses the expression levels of LDHA following its reintroduction. **C** LDH activity evaluated by measuring LDHA activity with an LDH assay kit after reintroduction. **D** Intracellular lactate levels measured to assess changes after LDHA re-expression. **E** Intracellular lactate content measured to evaluate changes in lactate levels after re-expression of SP1 and c-MYC. **F**, **G** Overexpression of LDHA in TAB182 knockdown cells increases cellular radioresistance. Data represent means ± SDs from three independent experiments. *P < 0.05; **P < 0.01; ***P < 0.001.

To further determine whether the metabolic alterations induced by TAB182 knockdown are due to the decreased expression of LDHA, LDHA rescue experiments were performed in TAB182 knockdown cell lines. The changes in relevant metabolic phenotypes were examined. Western blot experiments confirmed the efficiency of LDHA rescue (Fig. 5A, B). Subsequently, lactate dehydrogenase (LDH) enzyme activity was assessed, and it was found that reintroducing LDHA in TAB182 knockdown cell lines could reverse the decreased LDH enzyme activity observed after TAB182 knockdown (Fig. 5C). Additionally, intracellular lactate levels were examined, and reintroducing LDHA reversed the decreased lactate levels caused by TAB182 knockdown (Fig. 5D), indicating that TAB182 does affect intracellular lactate production by influencing LDHA expression. Furthermore, subsequent to utilizing SP1 and c-MYC to facilitate LDHA expression (Fig. 4G, H), a reversal of the decreased intracellular lactate production induced by TAB182 knockdown was observed (Fig. 5E). This indicates that TAB182 influences LDHA expression by regulating the expression of LDHA transcription factors, thereby affecting LDHA expression and subsequently impacting tumor cell glucose metabolism. Finally, through colony formation assay, it was found that replenishing LDHA in TAB182 knockdown cells could reverse the increased radiation sensitivity induced by TAB182 knockdown (Fig. 5F, G). This further substantiates that TAB182 influences cellular radiation sensitivity by affecting cellular metabolism through LDHA.

### TAB182 is related to the dismal prognostic outcome of cancer patients

**Fig. 6:**
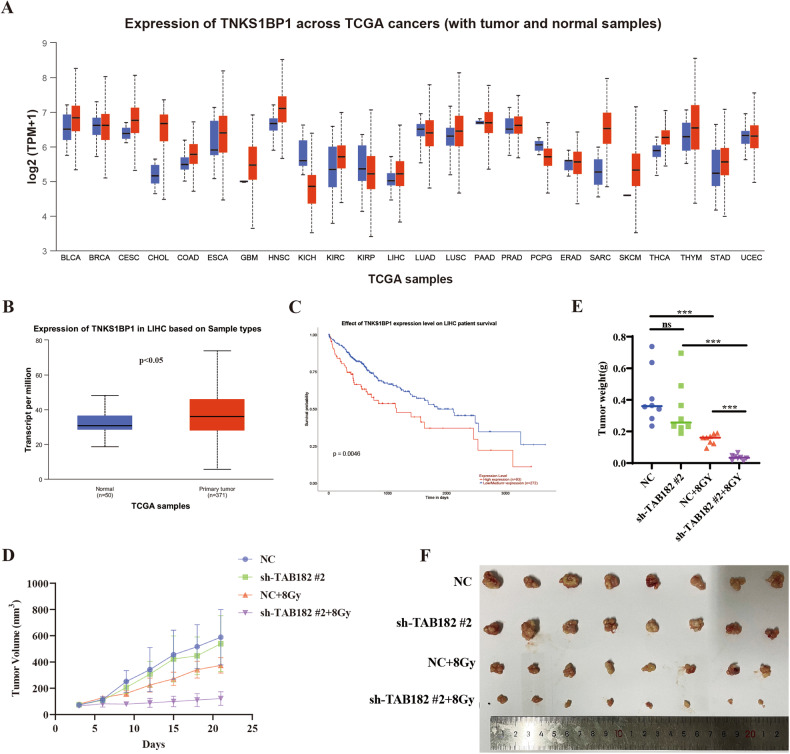
TAB182 and prognosis in cancer patients. **A** Analysis of the TCGA database indicates high TAB182 expression in multiple cancer tissues. **B**, **C** TAB182 is highly expressed in liver cancer tissues and negatively associated with cancer patient prognosis. **D** The tumor sizes in each group were tracked every 3 days, starting from the first day following radiation therapy. **E**, **F** Tumor size and weight in each group after 21 days of radiation treatment. Data represent means ± SDs from three independent experiments. *n* = 8, *P < 0.05; **P < 0.01; ***P < 0.001.

Furthermore, TAB182 expression in various cancer tissues in the TCGA public database was analyzed using the UCLA website (https://ualcan.path.uab.edu). The results indicated that TAB182 is predominantly overexpressed in many tumors, such as liver cancer, breast cancer, and head and neck squamous cell carcinoma, among others (Fig. 6A). It was also noted that TAB182 upregulation was negatively associated with prognostic outcomes in cancer patients. For instance, in liver cancer patients, TAB182 expression was markedly upregulated compared to non-carcinoma tissue, and this was related to a worse prognosis for patients (Fig. 6B, C). This suggests that high TAB182 expression is associated with an unfavorable prognostic outcome in cancer patients. Therefore, xenograft tumor experiments were conducted to validate this. The results revealed that knocking down TAB182 alone did not significantly impact the tumor cell growth rate. However, following irradiation treatment, the TAB182 knockdown group exhibited a notably lower tumor cell growth rate than the control group, indicating an increase in the radiation sensitivity of tumor cells (Fig. 6D). Euthanasia of nude mice was performed to dissect and weigh tumor tissues. The results showed that knocking down TAB182 alone did not have a significant impact on tumor growth. Nevertheless, when combined with irradiation treatment, the tumor weight of the TAB182 knockdown group was evidently lower compared to the control group (Fig. 6E, F). These findings further support the hypothesis that TAB182 is linked to an unfavorable prognosis in cancer patients and that the knockdown of TAB182 enhances the radiation sensitivity of tumor cells.

## Discussion

Tumor cells undergo metabolic reprogramming to meet their rapid growth needs. The Warburg effect enables tumor cells to generate sufficient building blocks for growth and proliferation. Even under aerobic conditions, tumor cells utilize aerobic glycolysis for energy generation, producing abundant intermediate metabolites [20]. These metabolites are then used in further metabolic processes to synthesize fatty acids, nucleotides, amino acids, and other biomolecules, providing essential raw materials for tumor cell growth and reproduction [21]. Metabolic reprogramming is a characteristic feature of tumor cells [22]. Tumor cells undergo metabolic changes in response to stimuli, ensuring their survival needs, such as during chemotherapy and radiotherapy. In chemoresistant tumor cells, various changes occur in lipid metabolism, including enhanced de novo synthesis of fatty acids and cholesterol, increased uptake and β-oxidation of fatty acids, promotion of lipid storage and mobilization in lipid droplets, and upregulation of several key enzymes and regulatory factors in lipid metabolism [23]. Chemotherapeutic drugs can induce metabolic compensations in tumor cells, leading to drug resistance. For instance, platinum-based drugs increase glutamine metabolism, doxorubicin enhances the pentose phosphate pathway, and paclitaxel boosts mitochondrial activity [24]. Radiation therapy can also induce changes in tumor cell metabolic pathways, leading to radiation resistance [13, 25]. Research has shown that radiation therapy triggers the Warburg effect in tumor cells, characterized by glycolysis under aerobic conditions. There is a close interaction of cellular metabolism with DNA damage response [26]. Reactive oxygen species, a result of metabolic reactions, can cause DNA oxidative damage [27]. Conversely, the antioxidant glutathione relies on metabolic pathways for its production [28]. Metabolic enzymes and metabolites actively participate in the repair of DNA double-strand breaks (DSBs). For example, pyruvate kinase M2 (PKM2), a central enzyme in glycolysis, undergoes phosphorylation by ATM, facilitating its role in homologous recombination repair of DSBs [29]. Consequently, metabolic changes induced by radiation may correlate with the radiation sensitivity of tumor cells. Studies have shown a close association between glucose metabolism and radioresistance [30]. Radioresistant tumor cells often exhibit increased glucose metabolism. Targeted interventions on key molecules such as GLUT1, lactate, and LDHA are promising for augmenting radiosensitivity [31]. Additionally, activation of HIF-1 enhances glucose metabolism enzymes, triggering autophagy and angiogenesis, thereby contributing to radiation resistance [31]. Decreased mitochondrial oxidative stress and enhanced mitochondrial membrane potential promote DNA damage repair and inhibit apoptosis, leading to radiation resistance [32]. Notably, mitochondrial-associated proteins like SIRT3 and ATAD3A play crucial roles in the development of radioresistance [33, 34]. These findings suggest a strong link between tumor metabolic reprogramming and drug resistance. Hence, exploring tumor cell metabolic reprogramming is vital for understanding tumor pathogenesis and progression and for advancing cancer therapies.

Tumor cells display a higher glycolysis rate compared to healthy tissue cells. Lactate, a glycolytic byproduct once considered metabolic waste, is now understood to re-enter the TCA cycle, supplying energy to cells [35, 36]. The exact pathway for lactate re-entry remains unclear. Lactate has been shown to alter the acidic tumor microenvironment, inhibiting cellular immunity and facilitating tumor immune escape [37]. Additionally, it enhances tumor cell invasion and migration [38]. Lactate production through glycolysis can induce radiation resistance in tumor cells, promoting tumor growth [11]. Lactate also affects tumor cell radiation sensitivity by influencing DNA damage repair [39]. LDHA, a key enzyme in lactate metabolism, catalyzes the conversion of pyruvate to lactate [38] and is significant in tumor cell radiation sensitivity [31]. High LDHA expression in tumors is linked to increased radiation resistance [40]. Therefore, investigating lactate production may offer a breakthrough in tumor therapy.

TAB182, a binding protein of tankyrase 1, negatively correlates with postoperative radiotherapy prognosis in esophageal cancer patients [41]. Esophageal cancer cells with elevated TAB182 expression demonstrate stronger radiotherapy resistance [14]. Overexpression of TAB182 increases cellular radioresistance by upregulating G2-M phase arrest [14] and is associated with repairing DNA double-strand breaks [42]. TAB182 promotes DNA-PKcs autophosphorylation, a key step in DNA double-strand break repair [19], and its downregulation leads to delayed clearance of γ-H2AX. TAB182 is a potential biomarker for screening and targeted therapy in diseases like esophageal cancer and plays a crucial role in resistance to radiotherapy and chemotherapy. However, research on TAB182 in tumors is still evolving, and its precise molecular mechanisms require further investigation.

This study reveals that radiation induces an increase in TAB182 expression, leading to elevated LDHA expression and increased lactate production. Reducing TAB182 in tumor cells decreases lactate production, thereby enhancing their radiation sensitivity. Further investigation shows that TAB182 influences the expression of transcription factors c-MYC and SP1, affecting LDHA expression and lactate production. This study is the first to identify TAB182 as a pivotal molecule influencing LDHA expression, a key enzyme in lactate metabolism. The findings highlight that reducing TAB182 can improve radiation sensitivity in tumor cells, offering a new approach to overcome radioresistance in tumor therapy.

## Materials and methods

### Cell culture

Human breast cancer MCF7 cells and human liver cancer HepG2 cells, purchased from the American Type Culture Collection (ATCC), were cultured in DMEM (HyClone) supplemented with 10% FBS (HyClone and PAN) and 1% penicillin-streptomycin. Cells were incubated in a humidified incubator at 37 °C with 5% CO_2_. For irradiation, cells underwent exposure to 60Co γ-rays at a dose rate of 85.69 cGy min^–1^ at ambient temperature.

### Plasmid and shRNA

Full sequences of TAB182, LDHA, c-MYC, and SP1 were cloned into the pcDNA3.1 vector to generate overexpression plasmids.

For silencing experiments, control, and TAB182-shRNA sequences were packaged into the lentiviral vector LV3 (H1/GFP&Puro) by GenePharma and used to infect MCF7 and HepG2 cells. Stable transfectants were selected after seven days of treatment with two µg/mL puromycin. TAB182-shRNA sequences are as follows:

The sequences of TAB182-shRNA are shown below:

TAB182-shRNA#1:

Sense: 5′-UAUCCAAGCGCUCUUCCCAAACUCC-3′

Antisense: 5′-GGAGUUUGGGAAGAGCGCUUGGAUA-3′

TAB182-shRNA#2:

Sense: 5′-AAGACGAGGAGUAAUCUUCACCCUG-3′

Antisense: 5′-CAGGGUGAAGAUUACUCCUCGUCUU-3′.

### RNA extraction and qPCR analysis

Total RNA was extracted using TRIzol reagent. One microgram of RNA was used to synthesize cDNA with HiScript II Q Select RT SuperMix for qPCR (+gDNA wiper) from Vazyme (Code No. R233–01) following specific protocols. For qPCR, 1 µL of cDNA was amplified using Taq Pro Universal SYBR qPCR Master Mix from Vazyme (Code No. Q712–02). β-actin served as the endogenous control. Primer sequences for qRT-PCR were as follows: β-actin: Forward: 5′- CATGTACGTTGCTATCCAGGC-3′, Reverse: 5′- CTCCTTAATGTCACGCACGAT-3′; for LDHA: Forward: 5′- TGGAGATTCCAGTGTGCCTG-3′; Reverse: 5′- TAGCCCAGGATGTGTAGCCT-3′; for LDHB: Forward: 5′- ACCAGTTGCGGAAGAAGAGG-3′; Reverse: 5′- CTCCCATGCTGCAGATCCAT-3′.

### Intracellular lactate, pyruvate, and ATP content detection

Lactate and pyruvate levels in cells were measured using Abbkine’s lactate assay kit (KTB1100) and pyruvate assay kit (KTB1121), respectively. The intracellular ATP content was determined using Biyuntian’s ATP assay kit (S0026). Cells were harvested in a culture dish and rinsed three times with pre-chilled phosphate-buffered saline (PBS) at 3000 rpm for 5 min. Afterward, the cells were resuspended in 1 mL of pre-chilled PBS for cell count determination. For each experimental group, 5 × 10^5^ cells were used. Subsequent experiments followed the instructions provided in the assay kit manuals.

### Lactate dehydrogenase activity assay

The intracellular LDH activity was assessed using Abbkine’s LDH activity assay kit (KTB1110). After harvesting in a culture dish, cells were washed three times with pre-chilled PBS at 3000 rpm for 5 min. Following the wash, the cells were resuspended in 1 mL of pre-chilled PBS for cell count determination. For each experimental group, 5 × 10^5^ cells were utilized. Subsequent experiments adhered to the instructions outlined in the assay kit manual.

### Western blot analysis and antibodies

RIPA buffer containing Protease Inhibitor Cocktail (Selleck, B14001) was used for cell lysis. After centrifuging the cell extracts at 12,000 × *g* for 15 min, the supernatant was collected. Approximately 40 µg of total proteins were separated by SDS-polyacrylamide gel electrophoresis before being transferred to nitrocellulose (NC) membranes. Following a 2-h blocking step with 5% defatted milk at room temperature, the membranes were subsequently probed with primary antibodies. Subsequently, the membranes were washed three times and further probed with horseradish peroxidase (HRP)-labeled secondary antibodies (diluted at 1:4000) for 1 h at room temperature, and protein bands were visualized using SuperSignal™ West Pico Plus Chemiluminescent Substrate (ThermoFisher Scientific, TL275133). The primary antibodies used were as follows: anti-TAB182 (Santa Cruz, sc-514517, 1:1000), anti-LDHA (Cell Signaling Technology, 3582, 1:1000), anti-LDHB (Proteintech, 14824–1-AP, 1:1000), anti-c-MYC (Cell Signaling Technology, 18583, 1:1000), anti-SP1 (Cell Signaling Technology, 9389, 1:1000), anti-α-tubulin (Santa Cruz, sc-69969, 1:1000), and anti-actin (Proteintech, 81115–1-RR, 1:1000). The enhanced chemiluminescent reagent (Thermo, MA, USA) was used to detect protein bands.

### mRNA stability assay

To assess RNA stability, cells were treated with actinomycin D (Santa Cruz) at a concentration of 5 ug/mL for specified time intervals. Following treatment, cells were harvested to isolate RNA samples for qPCR analysis.

### Dual-luciferase reporter assay

The DNA sequence of the LDHA promoter, spanning 2000 bp upstream, was inserted into the pGL-Basic firefly luciferase plasmid to create the LDHA promoter-reporter gene. The pRL-TK Renilla luciferase plasmid was used as the endogenous reference reporter gene. The experiment was conducted using the Nano-Glo® Dual-Luciferase® Reporter Assay System kit (E1910) from Promega, following the instructions outlined in the assay kit manual.

### Colony formation assay

MCF7 (NC, sh-TAB182 #1, sh-TAB182 #2) and HepG2 (NC, sh-TAB182 #1, sh-TAB182 #2) cells were collected and seeded into six-well plates at various specific cell densities until they adhered. The cell medium was changed every 4 days. After a 14-day incubation period, cell colonies, each containing approximately 50 cells, were fixed in 4% paraformaldehyde and then stained using a 1× crystal violet staining solution. The colonies were subsequently rinsed with PBS, and their count was determined through visual observation. The colony formation rate was calculated as follows: (number of cell colonies in each well/number of cells planted per well) × 100%. PS: Number of cells planted per well (HepG2: 0 Gy 300 cell/well, 4 Gy 1000 cell/well, 8 Gy 2000 cell/well, MCF7: 0 Gy 500 cell/well, 4 Gy 1500 cell/well, 8 Gy 3000 cell/well).

### Xenograft tumor model in nude mice

Male BALB/c-Nude mice aged 4 weeks were purchased from SPF (Beijing) Biotechnology Co. Ltd. The mice were divided into four randomized groups (8 mice/group): HepG2-NC, HepG2-sh-TAB182, HepG2-NC + radiation, and HepG2-sh-TAB182 + radiation groups. They were subcutaneously injected with either TAB182 knockout HepG2 cells or wild-type HepG2 cells (1 × 10^7^ cells/0.1 mL/mouse) according to their respective groups. Three days after cell injection, the mice received a local radiation dose of 8 Gy. Starting from the day of radiation, tumor width (*W*) and length (*L*) were measured using calipers at 3-day intervals to monitor tumor growth. Four weeks after cell injection, the mice were euthanized, and tumor tissue was extracted, weighed, and photographed. The experimental procedure and the use of animals were approved by the Institutional Animal Care and Use Committee of the Laboratory Animal Center. All animal experiments were conducted in accordance with the Control of Animal Laboratory of the Academy of Military Medical Sciences and adhered to the principles of animal research (IACUC-DWZX-2023–535).

### Statistical analysis

The results were presented as mean ± SEMs or SDs. Statistical analysis was performed using GraphPad Prism 9 (GraphPad Software, Inc.). Between-group differences were analyzed using an unpaired two-tailed Student’s *t* test (*P* < 0.05 indicated significance). One-way analysis of variance with multiple comparison tests was conducted to compare three or more groups (*P* < 0.05 indicated statistical significance).

### Supplementary information

Original data-WB

Original data-qPCR

Supplement Figure

aj-checklist

## Data Availability

The datasets generated during the current study are available from the corresponding author upon reasonable request.
