# Supplementary material for: TAB182 regulates glycolytic metabolism by controlling LDHA transcription to impact tumor radiosensitivity
Source: Cell Death Dis. 2024 Mar 13;15(3):209. doi: 10.1038/s41419-024-06588-8 (PMC10937931; doi:10.1038/s41419-024-06588-8)

Figure. 1A - 1

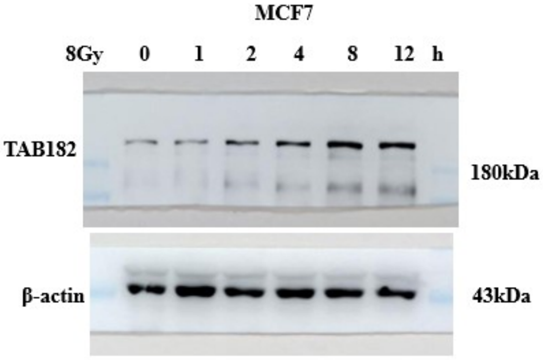

Figure. 1B - 1

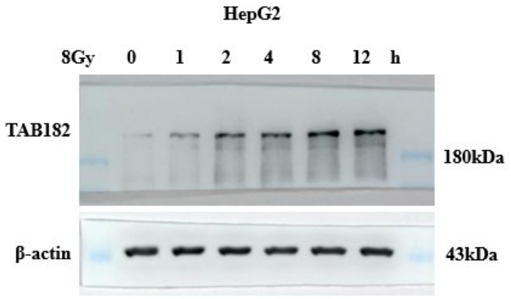

Figure. 1A – 2

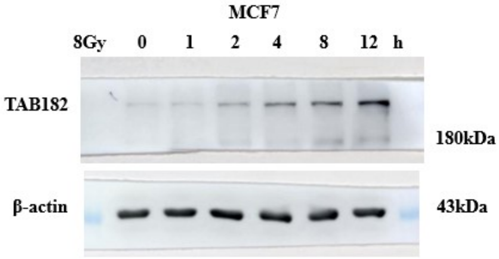

Figure. 1B – 2

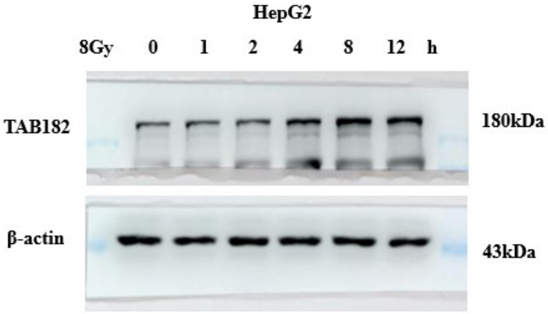

Figure. 1A – 3

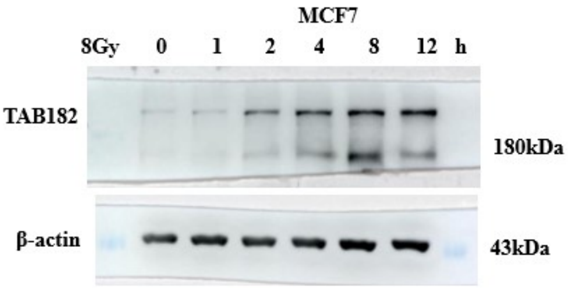

Figure. 1B – 3

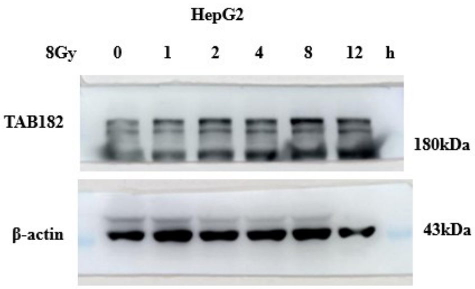

Figure. 1C – 1

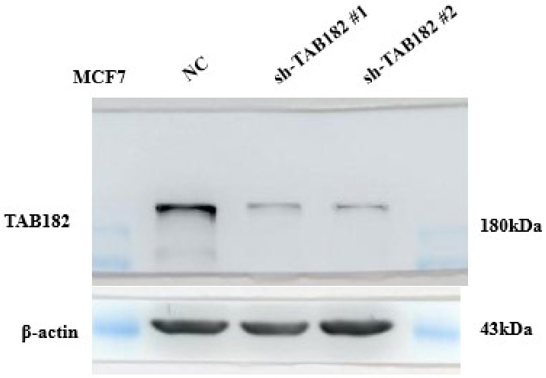

Figure. 1D – 1

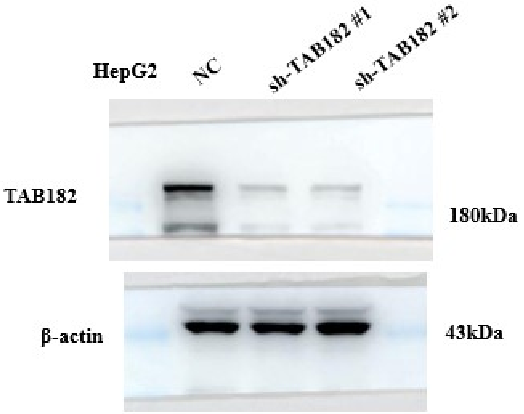

Figure. 1C – 2

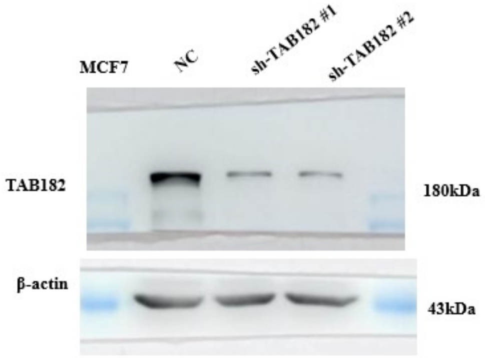

Figure. 1D – 2

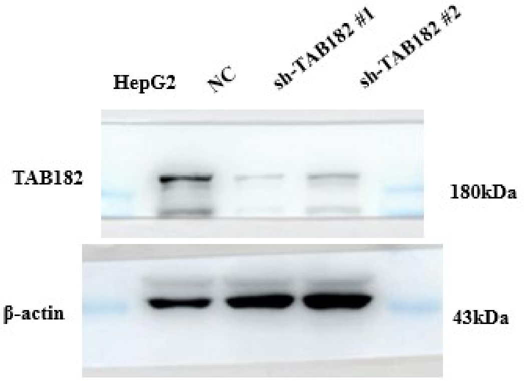

Figure. 1C – 3

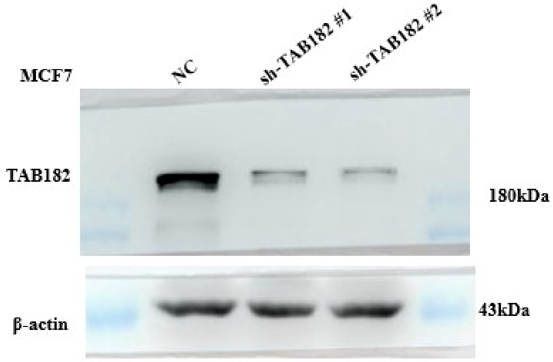

Figure. 1D – 3

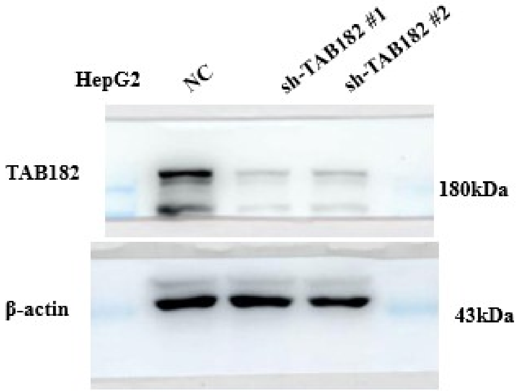

Figure. 1G – 1

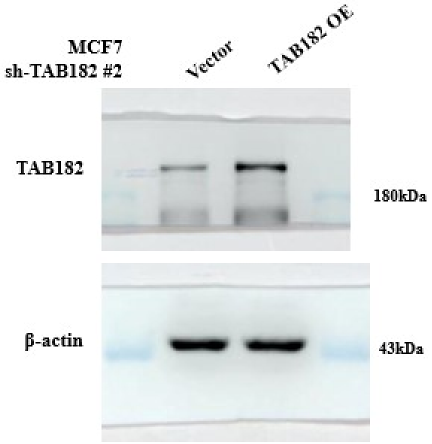

Figure. 1H– 1

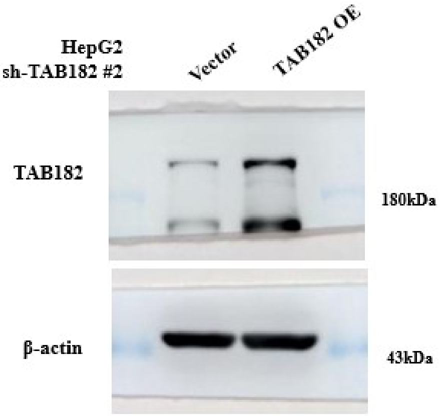

Figure. 1G – 2

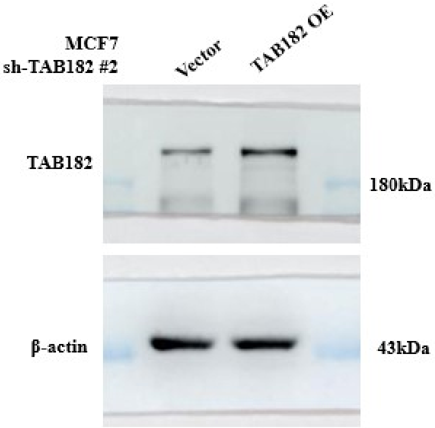

Figure. 1H– 2

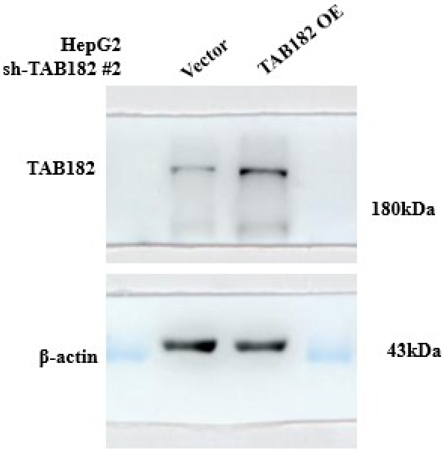

Figure. 1G – 3

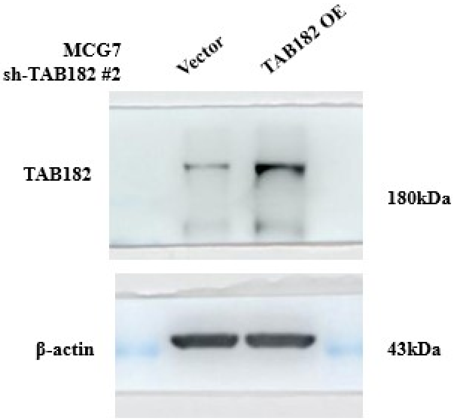

Figure. 1H– 3

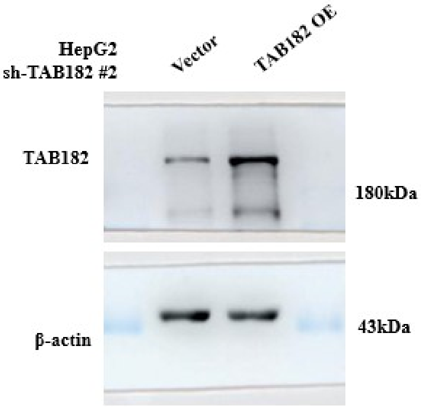

Figure. 3E – 1

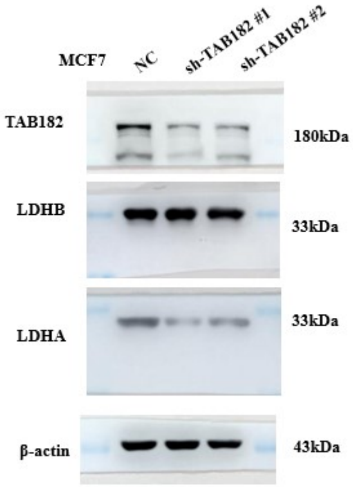

Figure. 3F– 1

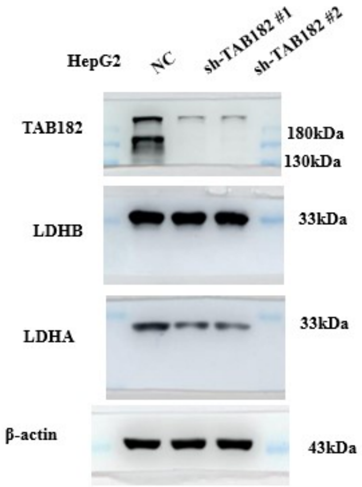

Figure. 3E – 2

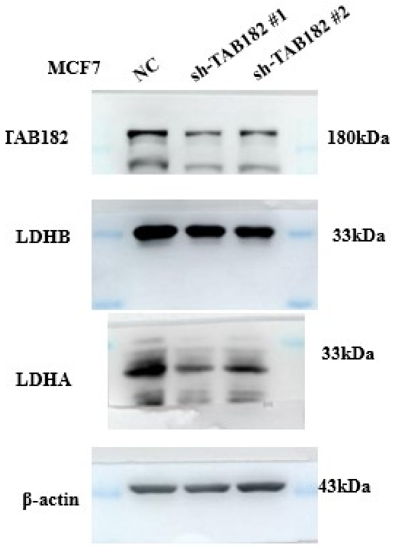

Figure. 3F– 2

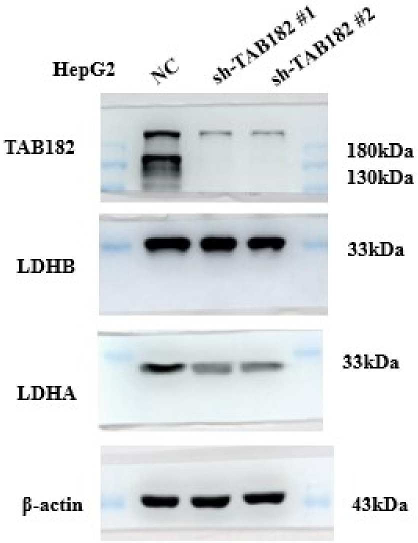

Figure. 3E – 3

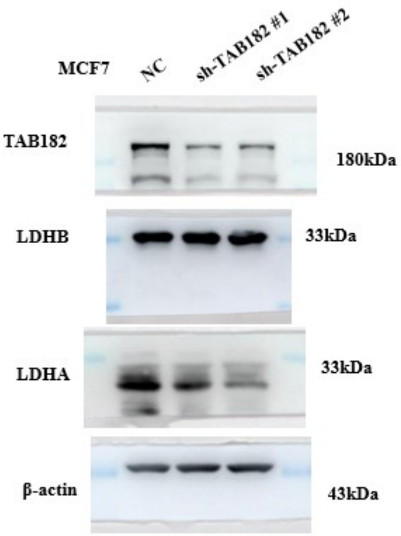

Figure. 3F–3

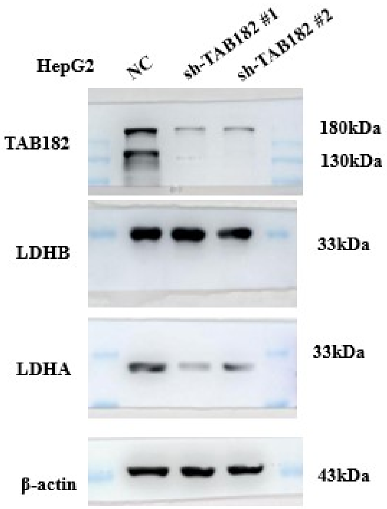

Figure. 3G – 1

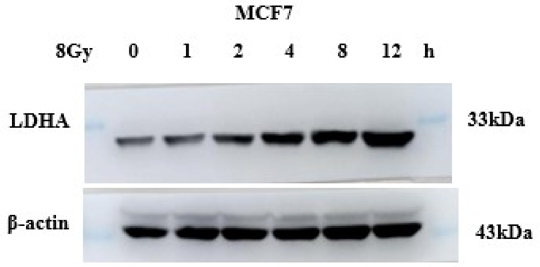

Figure. 3G – 2

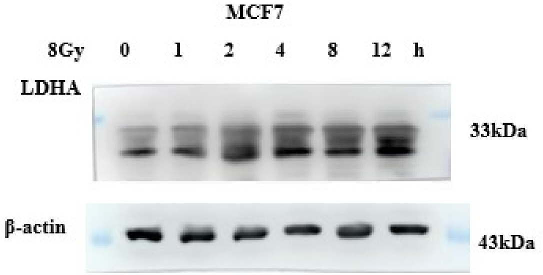

Figure. 3G – 3

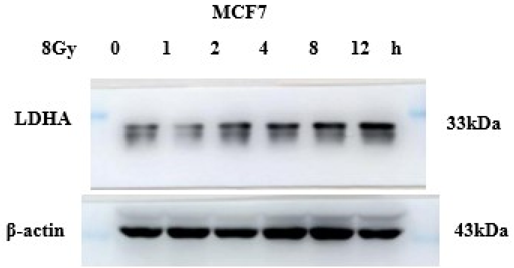

Figure. 3H– 1

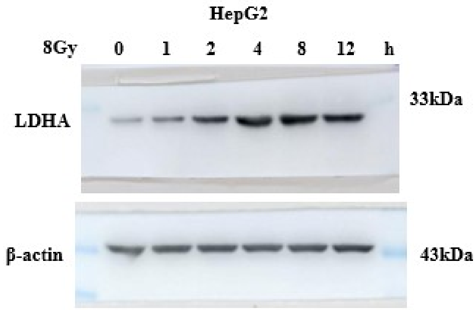

Figure. 3H– 2

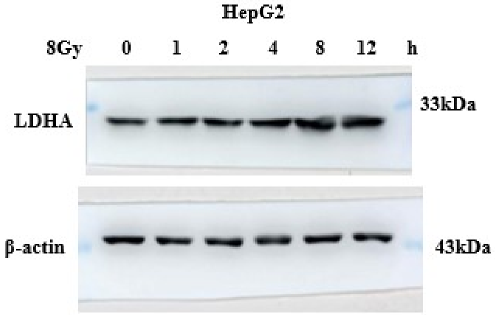

Figure. 3H– 3

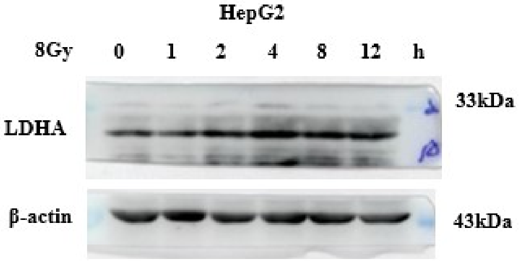

Figure. 3I – 1

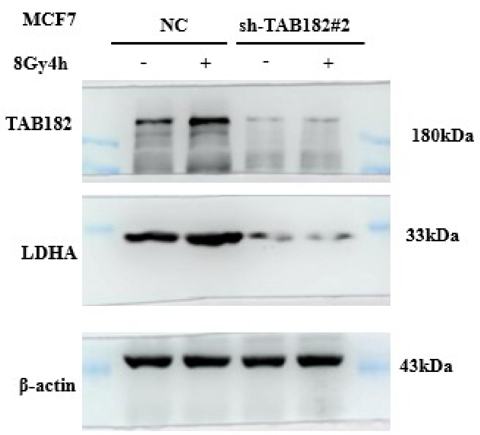

Figure. 3J– 1

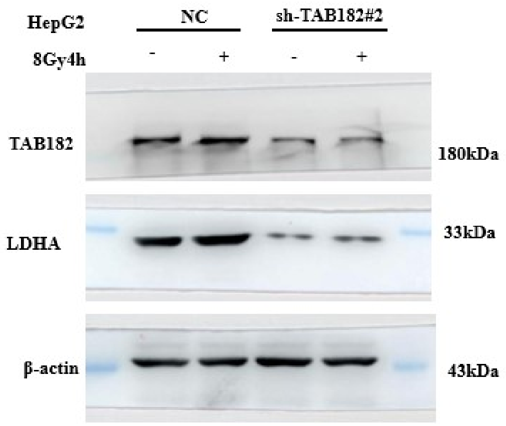

Figure. 3I – 2

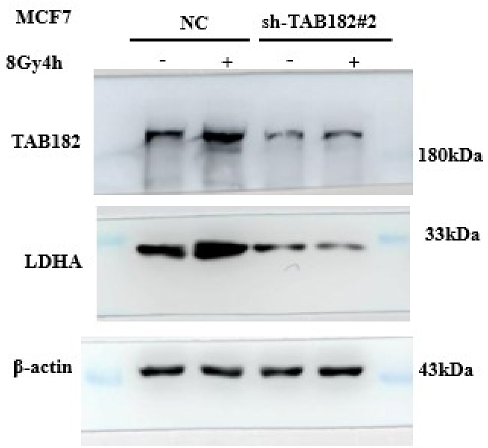

Figure. 3J– 2

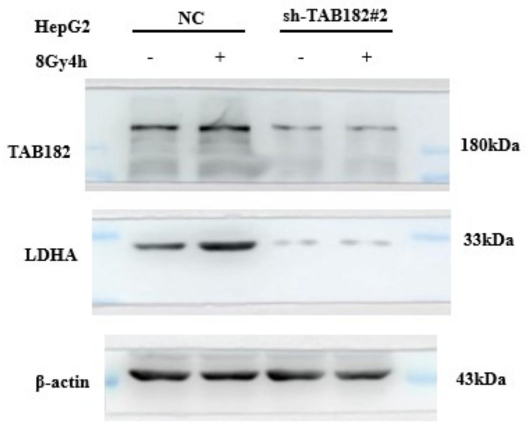

Figure. 3I – 3

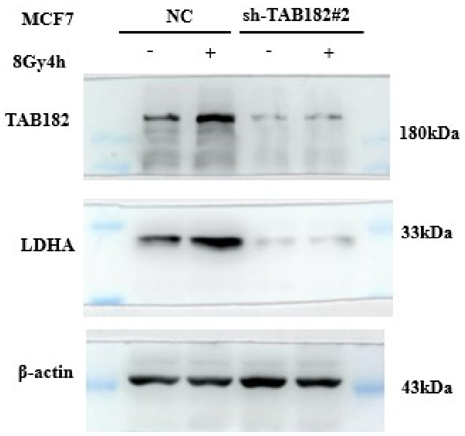

Figure. 3J– 3

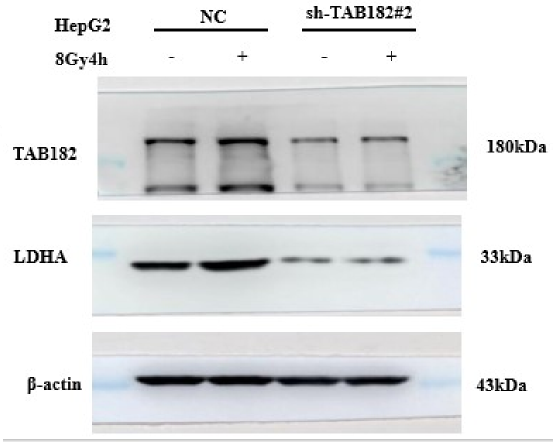

Figure. 4D – 1

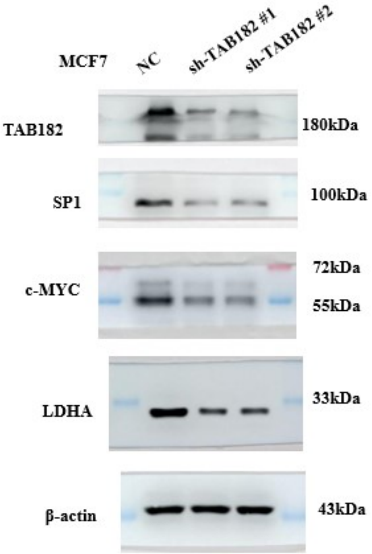

Figure. 4D – 2

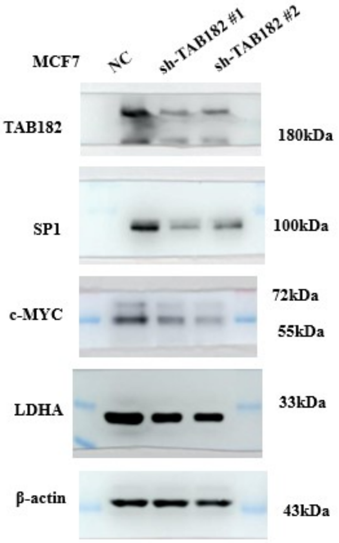

Figure. 4D – 3

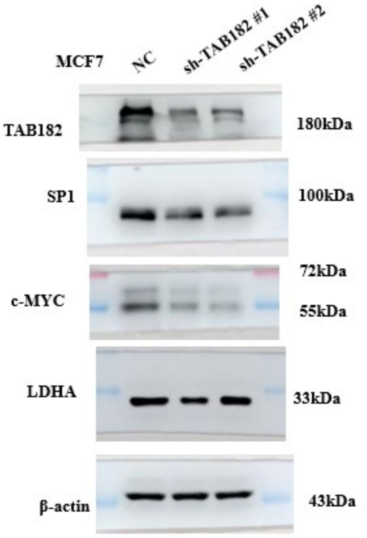

Figure. 4E– 1

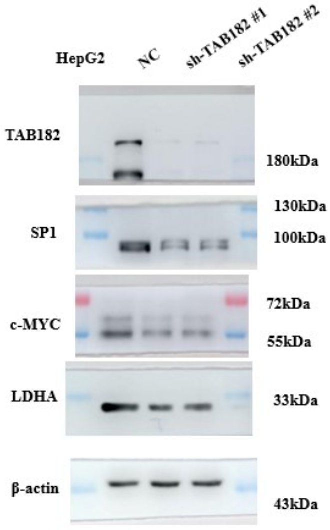

Figure. 4E– 2

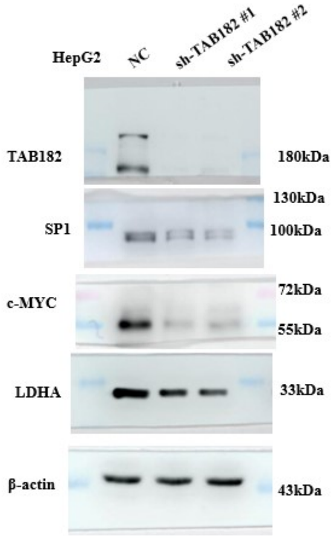

Figure. 4E– 3

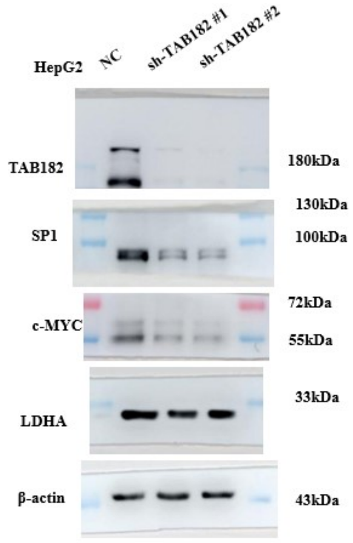

Figure. 4G – 1

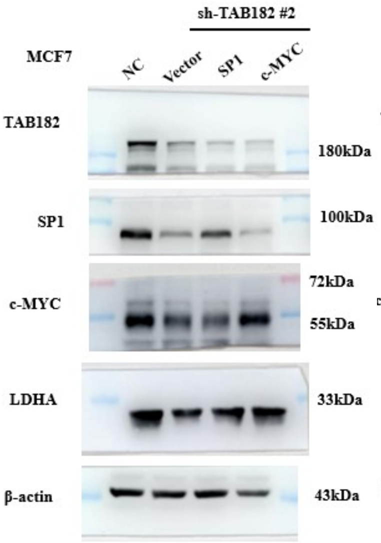

Figure. 4G – 2

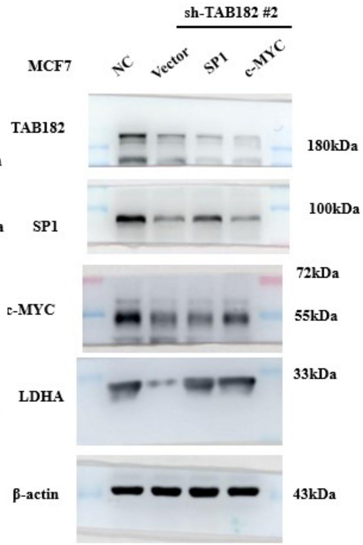

Figure. 4G – 3

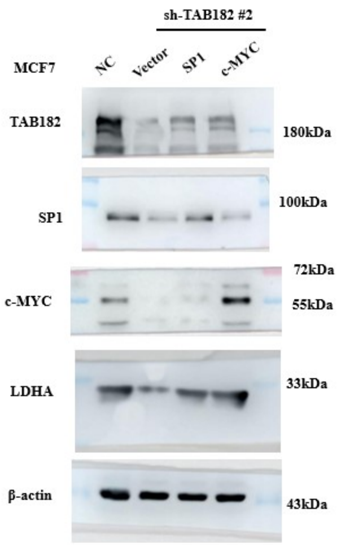

Figure. 4H– 1

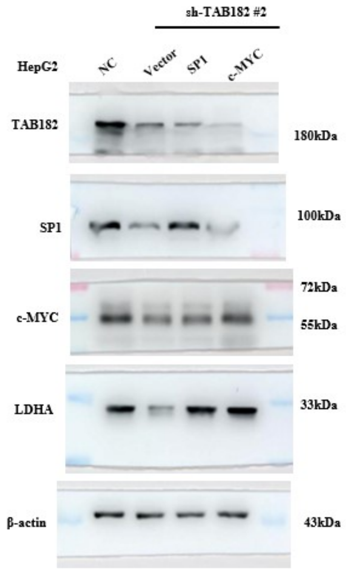

Figure. 4H– 2

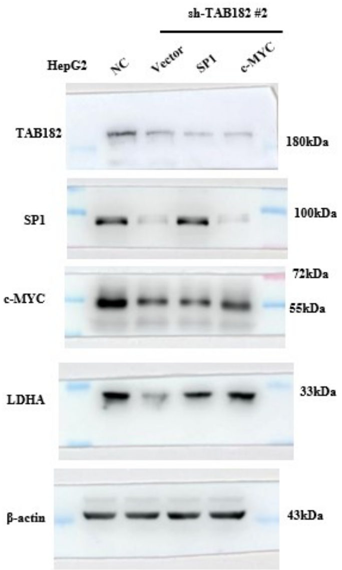

Figure. 4H– 3

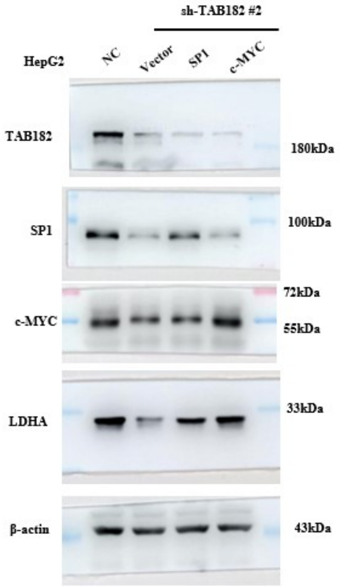

Figure. 5A – 1

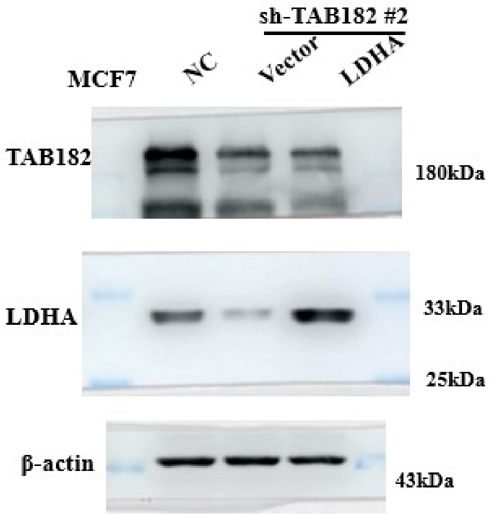

Figure. 5B – 1

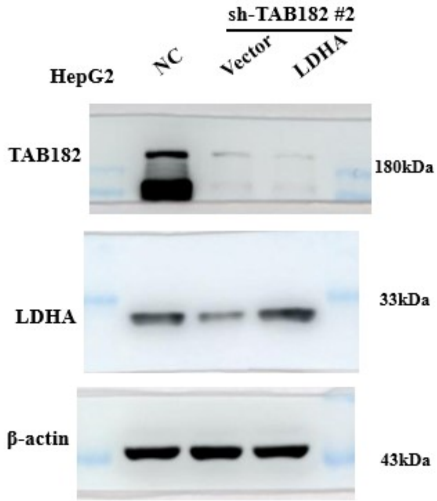

Figure. 5A – 2

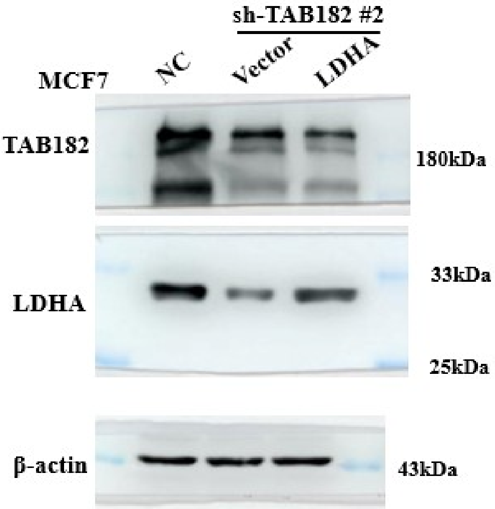

Figure. 5B – 2

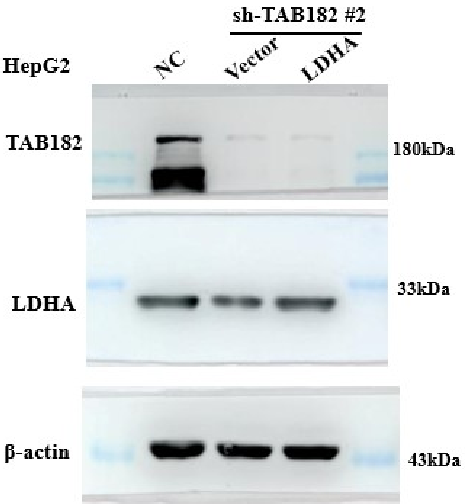

Figure. 5A – 3

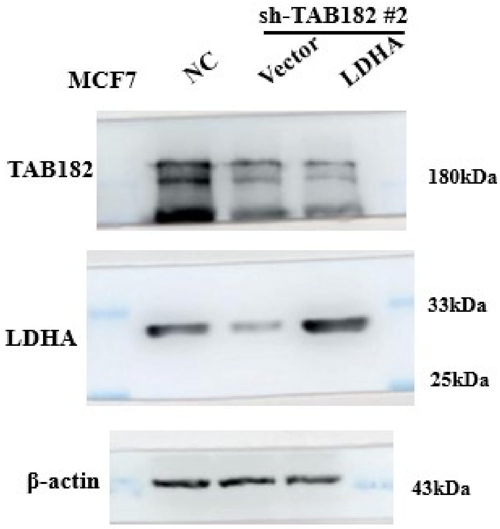

Figure. 5B – 3

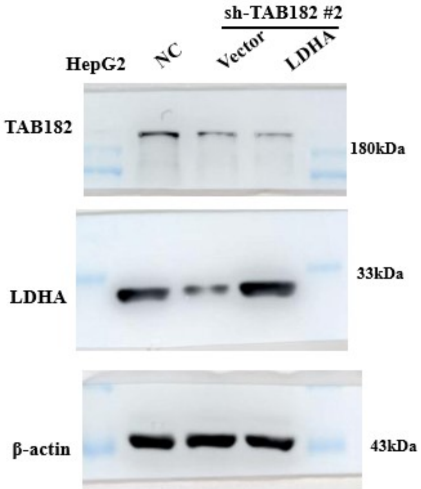

Supplementary Information. E – 1

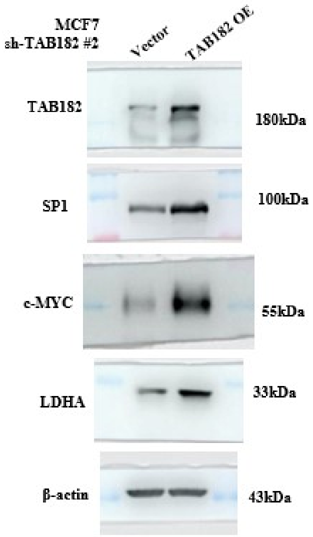

Supplementary Information. E –2

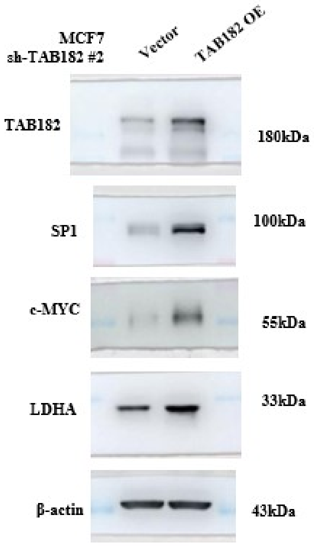

Supplementary Information. E – 3

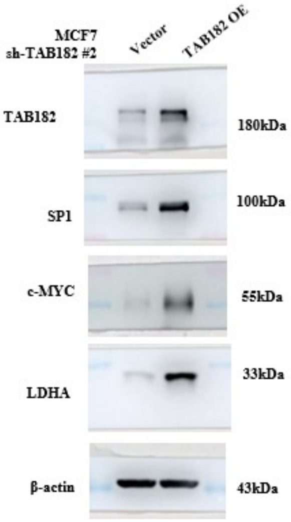

Supplementary Information. F – 1

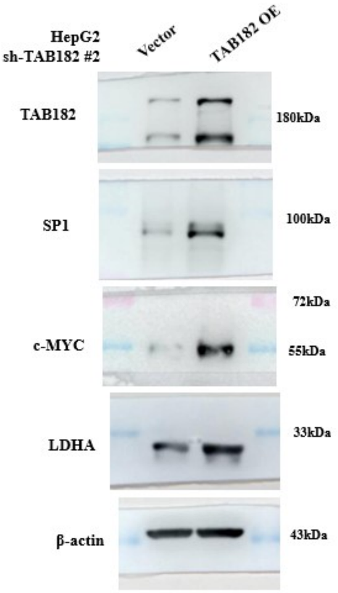

Supplementary Information. F – 2

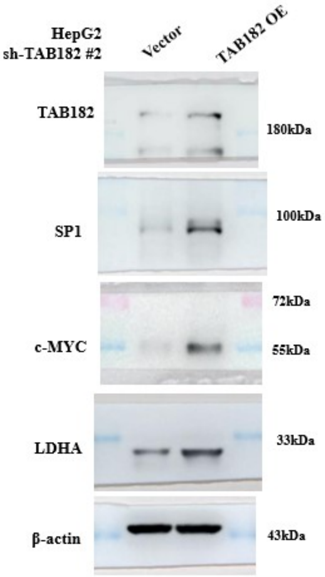

Supplementary Information. F – 3

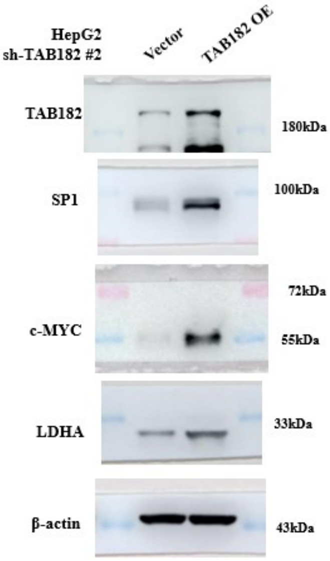

Supplement: Supplementary file 1 — Original data-WB [file 41419_2024_6588_MOESM1_ESM.pdf]
