# Supplementary material for: TAB182 regulates glycolytic metabolism by controlling LDHA transcription to impact tumor radiosensitivity
Source: Cell Death Dis. 2024 Mar 13;15(3):209. doi: 10.1038/s41419-024-06588-8 (PMC10937931; doi:10.1038/s41419-024-06588-8)
Supplement: Supplementary file 2 — Original data-qPCR [file 41419_2024_6588_MOESM2_ESM.pdf]

MCF7	Target	Sample	Cq Mean	Δct	ΔΔct	POWER
	182	nc	25.28	5.995199795		1
	182	#1	26.60	7.723205174	1.728005379	0.301869
	182	#2	25.76	9.631008485	3.63580869	0.080447
	actin	nc	19.28			
	actin	#1	18.88			
	actin	#2	16.13			
	182	nc	25.12	5.9644709		1
	182	#1	27.36	8.712523855	2.748052955	0.148852
	182	#2	26.27	10.1501579	4.185686995	0.054952
	actin	nc	19.15			
	actin	#1	18.65			
	actin	#2	16.12			
	182	nc	25.08	6.073634594		1
	182	#1	27.30	8.688215325	2.614580731	0.16328
	182	#2	26.16	10.46185395	4.388219361	0.047755
	actin	nc	19.01			
	actin	#1	18.61			
	actin	#2	15.70			

MCF7	Target	Sample	Cq Mean	Δct	ΔΔct	POWER
	LDHA	M NC	17.57	-0.136558748		1
	LDHA	M #1	17.95	0.385373436	0.521932184	0.696438477
	LDHA	M #2	18.17	0.862170442	0.998729189	0.500440623
	ACTIN	M NC	17.71			
	ACTIN	M #1	17.57			
	ACTIN	M #2	17.30			
	LDHA	M NC	17.52	-0.58935915		1
	LDHA	M #1	17.71	0.626616955	1.215976106	0.430481724
	LDHA	M #2	18.38	0.893110994	1.482470144	0.357875542
	ACTIN	M NC	18.11			
	ACTIN	M #1	17.08			
	ACTIN	M #2	17.49			
	LDHA	M NC	17.55	-0.836590489		1
	LDHA	M #1	18.29	0.222079738	1.058670227	0.480074354
	LDHA	M #2	18.32	0.257429595	1.094020084	0.468454202
	ACTIN	M NC	18.38			
	ACTIN	M #1	18.07			
	ACTIN	M #2	18.07			

HepG2	Target	Sample	Cq Mean	Δct	ΔΔct	POWER
182	nc	26.40	19.23	7.168420544		1
182	#1	26.90	18.47	8.431635842	1.263215298	0.416614
182	#2	29.13	20.36	8.772497671	1.604077127	0.328946
ldha	nc	19.18	19.23	-0.051908369		1
ldha	#1	19.11	18.47	0.641827146	0.693735515	0.618251
ldha	#2	20.83	20.36	0.468447594	0.520355963	0.6972

actin	nc	19.23
actin	#1	18.47
actin	#2	20.36

182	nc	26.49	16.60	9.885129146		1
182	#1	27.05	15.60	11.45506058	1.569931436	0.336824
182	#2	29.20	15.75	13.4482467	3.563117553	0.084605
ldha	nc	19.09	16.60	2.484239669		1
ldha	#1	19.09	15.60	3.493589852	1.009350182	0.49677
ldha	#2	20.73	15.75	4.972689479	1.479099628	0.358713
actin	nc	16.60				
actin	#1	15.60				
actin	#2	15.75				

182	nc	26.49	19.00	7.483759199		1.00
182	#1	26.88	18.41	8.476187964	0.992428765	0.502631
182	#2	28.87	18.07	10.79821008	3.31445088	0.10052
ldha	nc	19.29	19.00	0.290730288		1
ldha	#1	19.48	18.41	1.069168435	0.778438147	0.582998
ldha	#2	18.91	18.07	0.83503154	0.544301252	0.685723
actin	nc	19.00				
actin	#1	18.41				
actin	#2	18.07				

MCF7 mRNA stability	Target	Sample	Cq Mean	Δct	ΔΔct	POWER
	LDHA MCF7	NC 0H	20.38	1.542295442		1
	LDHA MCF7	#2 0H	20.77	1.622517456		1
	LDHA MCF7	NC 4H	20.58	1.756448934	0.214153492	0.862052
	LDHA MCF7	#2 4H	21.02	1.907457923	0.284940467	0.820775
	LDHA MCF7	NC 8H	23.91	1.898531288	0.356235847	0.7812
	LDHA MCF7	#2 8H	21.21	2.018102279	0.395584823	0.760181
	LDHA MCF7	NC 12H	22.63	2.47636415	0.934068708	0.52338
	LDHA MCF7	#2 12H	22.15	2.702492265	1.079974808	0.473037
	LDHA MCF7	NC 24H	26.80	4.593190397	3.050894955	0.120667
	LDHA MCF7	#2 24H	26.29	4.968261419	3.345743963	0.098363
	actin MCF7	NC 0H	18.84			
	actin MCF7	#2 0H	19.14			
	actin MCF7	NC 4H	18.82			
	actin MCF7	#2 4H	19.12			
	actin MCF7	NC 8H	22.01			
	actin MCF7	#2 8H	19.19			
	actin MCF7	NC 12H	19.45			
	actin MCF7	#2 12H	20.16			
	actin MCF7	NC 24H	22.21			
	actin MCF7	#2 24H	21.32			

HepG2 mRNA stability	Target	Sample	Cq Mean	Δct	ΔΔct	POWER
	LDHA HepG2	NC 0H	21.47	1.694846191		1
	LDHA HepG2	#2 0H	21.17	1.883097023		1
	LDHA HepG2	NC 4H	21.59	1.891654422	0.196808231	0.872479
	LDHA HepG2	#2 4H	21.58	2.160303804	0.277206781	0.825187
	LDHA HepG2	NC 8H	23.37	2.173528572	0.478682381	0.717633
	LDHA HepG2	#2 8H	23.90	2.277394404	0.394297381	0.76086
	LDHA HepG2	NC 12H	24.05	2.673139308	0.978293117	0.50758
	LDHA HepG2	#2 12H	23.00	2.695756265	0.812659243	0.569331
	LDHA HepG2	NC 24H	32.37	3.956070045	2.261223854	0.208595
	LDHA HepG2	#2 24H	32.99	4.123234192	2.240137169	0.211666
	actin HepG2	NC 0H	19.78			

actin HepG2	#2 0H	19.29
actin HepG2	NC 4H	19.70
actin HepG2	#2 4H	19.61
actin HepG2	NC 8H	21.26
actin HepG2	#2 8H	20.44
actin HepG2	NC 12H	21.38
actin HepG2	#2 12H	21.38
actin HepG2	NC 24H	28.61
actin HepG2	#2 24H	26.87
