# Supplementary material for: TAB182 regulates glycolytic metabolism by controlling LDHA transcription to impact tumor radiosensitivity
Source: Cell Death Dis. 2024 Mar 13;15(3):209. doi: 10.1038/s41419-024-06588-8 (PMC10937931; doi:10.1038/s41419-024-06588-8)
Supplement: Supplementary file 3 — Supplement Figure [file 41419_2024_6588_MOESM3_ESM.pdf]

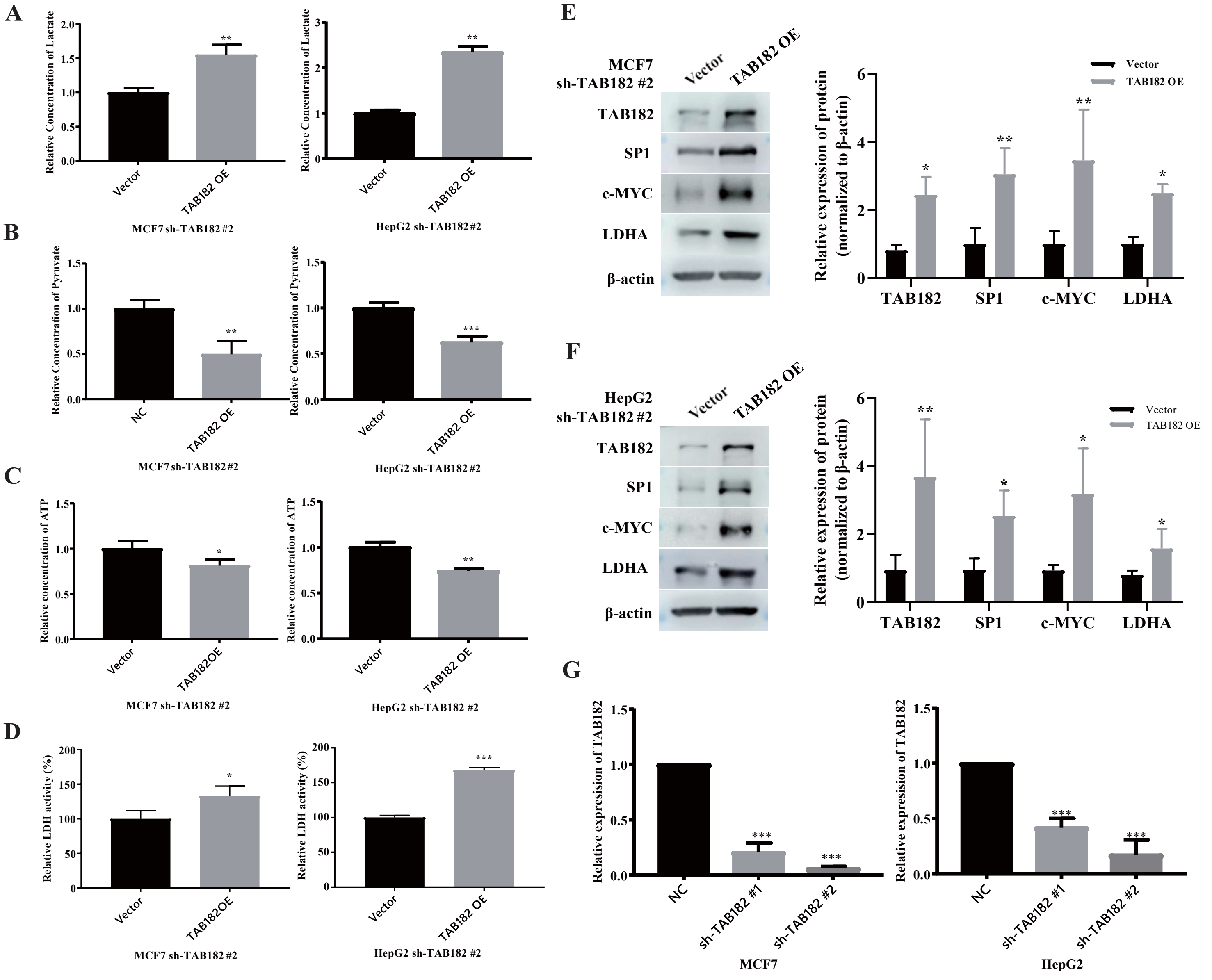


## Supplementary Information:

**A.** Overexpression of TAB182 increases intracellular lactate levels. **B.** Overexpression of TAB182 reduces intracellular pyruvate levels. **C.** Overexpression of TAB182 reduces intracellular ATP levels. **D.** Overexpression of TAB182 increases lactate dehydrogenase activity. **E and F.** Overexpression of TAB182 promotes SP1, c-MYC and LDHA expression. **G.** qPCR experiment to detect TAB182 knockdown efficiency. Data represent means ±SDs from three independent experiments. *P<0.05; **P<0.01; ***P<0.001
